# Towards Real-Time In-Situ Mid-Infrared Spectroscopic Ellipsometry in Polymer Processing

**DOI:** 10.3390/polym14010007

**Published:** 2021-12-21

**Authors:** Alexander Ebner, Robert Zimmerleiter, Kurt Hingerl, Markus Brandstetter

**Affiliations:** 1 RECENDT—Research Center for Non-Destructive Testing GmbH, 4040 Linz, Austria; alexander.ebner@recendt.at (A.E.); robert.zimmerleiter@recendt.at (R.Z.); 2 Center for Surface and Nanoanalytics, Johannes Kepler University, 4040 Linz, Austria; kurt.hingerl@jku.at

**Keywords:** ellipsometry, quantum cascade laser, in-line monitoring, polymer films, polymer processing, real-time, mid-infrared spectroscopy

## Abstract

Recent developments in mid-infrared (MIR) spectroscopic ellipsometry enabled by quantum cascade lasers (QCLs) have resulted in a drastic improvement in signal-to-noise ratio compared to conventional thermal emitter based instrumentation. Thus, it was possible to reduce the acquisition time for high-resolution broadband ellipsometric spectra from multiple hours to less than 1 s. This opens up new possibilities for real-time in-situ ellipsometry in polymer processing. To highlight these evolving capabilities, we demonstrate the benefits of a QCL based MIR ellipsometer by investigating single and multilayered polymer films. The molecular structure and reorientation of a 2.5 µm thin biaxially oriented polyethylene terephthalate film is monitored during a stretching process lasting 24.5 s to illustrate the perspective of ellipsometric measurements in dynamic processes. In addition, a polyethylene/ethylene vinyl alcohol/polyethylene multilayer film is investigated at a continuously varying angle of incidence (0${}^{\circ }$– 50${}^{\circ }$) in 17.2 s, highlighting an unprecedented sample throughput for the technique of varying angle spectroscopic ellipsometry in the MIR spectral range. The obtained results underline the superior spectral and temporal resolution of QCL ellipsometry and qualify this technique as a suitable method for advanced in-situ monitoring in polymer processing.

## 1. Introduction

For decades, polymer thin films have played an important role in many areas of modern life. In addition to classical applications, such as packaging, recent developments indicate promising new applications in the energy sector, for example, high-end coatings in batteries, membranes in fuel cells and especially organic electronics such as light emitting diodes, solar cells, sensors, bio-sensors and detectors [1,2,3,4,5,6,7,8,9,10,11]. The importance and necessity of energy efficient technologies—in which polymer thin films might be key elements—is notably emphasized by the current developments related to climate change. Since technological progress is typically closely linked to the advancement of analytical instrumentation, novel insights into known phenomena as well as the discovery of entirely unknown phenomena are often enabled by a leap in resolution—spatial, temporal or spectral. Recently, the application of modern mid-infrared (MIR) laser technology—namely the quantum cascade laser (QCL)—brought such decisive improvements to the already well-established and versatile measurement technique of spectroscopic ellipsometry [12].

Spectroscopic ellipsometry is a well-known non-destructive optical method and a particularly powerful analytical tool to precisely quantify a large set of material parameters. Its principle is based on the illumination of a sample with broadband polarized radiation and the subsequent measurement of the change in polarization after reflection on or transmission through the probed material. Contrary to simple intensity measurements, the actually measured quantity—the change in polarization—is typically derived by evaluating the *intensity ratios* of polarization components, which results in essential practical benefits. Besides the fact, that this method is hardly affected by instabilities of the light source, atmospheric absorption and ambient unpolarized stray light, ellipsometry enables *absolute measurements* without the need of any reference measurements [13]. The investigated change in polarization is typically represented by the amplitude ratio *and* the phase shift between two orthogonal polarization states—two parameters which ellipsometry measures simultaneously. Above all, by applying fundamental physics and data modeling, the measured phase shifts and amplitude ratios can be translated to much more comprehensible quantities such as layer thicknesses, optical properties (e.g., complex refractive index or dielectric function), anisotropy, roughness, geometry factors, defects, and quantum confinement effects of nanostructures [14].

A number of advanced applications of ellipsometry for the investigation of polymer thin films can be found in the ultraviolet and visible spectral range (UV-VIS). Such advanced applications, beyond the classical use for highly precise thickness measurements include studies on swelling, dilation, degradation, glass transitions, complex diffusion mechanisms and electrochemical processes [15,16]. Further, reported contributions not only cover a wide range of polymer systems but also sophisticated analytical methods for the extraction of thermodynamic parameters [17,18,19]. The benefits of UV-VIS ellipsometric in-situ measurements were impressively demonstrated by evaluating dynamic processes, for example, the aging of polymer thin films monitored in a streamlined ellipsometry procedure and phase transitions in polymer thin films monitored by means of temperature-dependent spectroscopic ellipsometry [20,21]. However, since the UV-VIS is just sensitive to electronic states and excitons, measurements in the infrared spectral range (IR)—in which molecular vibrations, free-charge-carrier and phonon absorptions are probed—can provide valuable and diverse information. Besides information about layer thicknesses, modeling of IR ellipsometry spectra therefore provides information on chemical and structural information, for example, anisotropic infrared dielectric functions and optical constants, which can be interpreted in terms of predominant molecular orientations within a thin film or layered material [22,23,24]. Using this technique, multilayered polymer films were characterized by simultaneously analyzing thickness, roughness, and chemical composition with respect to the temperature and pH changes of a washing solution, which impressively demonstrates that IR ellipsometry is capable of *simultaneously* evaluating chemical, physical and thermal properties of polymer films [25]. To date, however, commercial ellipsometric instrumentation for the IR is still based on conventional Fourier-transform infrared (FTIR) spectrometers and thermal light sources, which in turn leads to significant limitations in terms of signal-to-noise ratio (SNR) as well as temporal and spatial resolution.

The invention of modern broadband MIR laser sources (QCLs and most recently also MIR supercontinuum sources) and their application in various areas of IR spectroscopy including MIR ellipsometry has drastically enhanced the technological possibilities and previous limitations could finally be overcome [26,27,28,29,30,31,32,33,34,35,36]. The intrinsic properties of QCLs, that is, high brightness, highly polarized emission and narrow line widths combined with broadband tunability as well as the potential of essentially diffraction limited spot sizes, significantly differ from conventional thermal IR emitters. The first realization of a QCL based *spectroscopic* ellipsometry instrument in 2019 revealed an improved SNR by a factor of 290, which in turn led to a reduction in measurement time by a factor of 66,000 compared to a state-of-the art FTIR ellipsometer. Thus, high quality ellipsometric measurements in the MIR, which take more than 16 h with a conventional instrument are now feasible in less than 1 s [12]. With this technological advancement, a whole new set of potential applications for MIR spectroscopic ellipsometry has opened up, for example, real-time in-situ monitoring of dynamic processes.

In this contribution we highlight the emerging capabilities of QCL based MIR ellipsometry with a focus on polymer processing and attempt to initiate further research to exploit the full potential of this novel technique. We exemplarily demonstrate the measurement of the molecular structure and reorientation of a 2.5 μm thin biaxially oriented polyethylene terephthalate (BOPET) film during a stretching process, which took 24.5 s until the film tore. Additionally, we investigate a polyethylene/ethylene vinyl alcohol/polyethylene (PE/EVOH/PE) multilayer film at continuously varying angles of incidence (0${}^{\circ }$– 50${}^{\circ }$) in 17.2 s. This represents an unprecedented measurement speed and sample throughput for the—powerful but up to now very time-consuming—technique of varying angle spectroscopic ellipsometry in the MIR. The demonstrated experiments underline the superior spectral and temporal resolution of QCL ellipsometry and qualify this technique as a method for advanced in-situ monitoring in polymer processing.

## 2. Materials and Methods

### 2.1. Quantum Cascade Laser Based Spectroscopic Ellipsometry Setup

**Figure 1:**
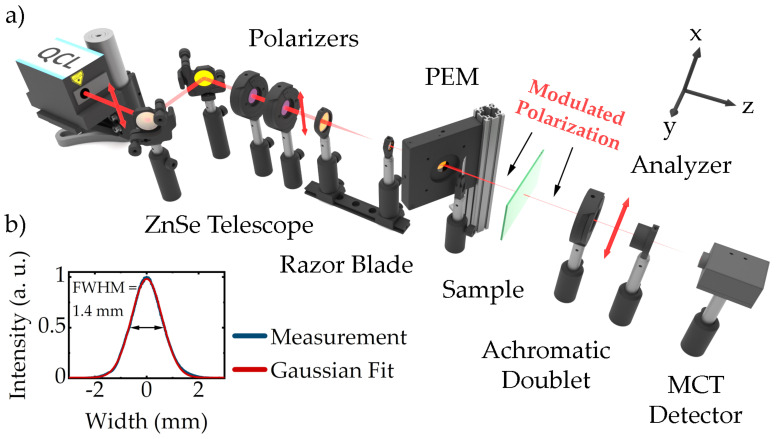
(**a**) Experimental Setup. The QCL radiation is guided through a set of two Si wire grid polarizers. Before the polarization state is modulated using a PEM, the beam waist is reduced. Thereby, reflections occurring inside the tilted PEM can be blocked by the subsequent razor blade. After interaction with the sample the radiation passes a third Si polarizer (analyzer) and is focused on an MCT detector by means of an achromatic doublet lens. (**b**) Beam waist at sample position measured with a microbolometer FPA at an exemplaric wavelength of 1052 cm^−1^.

The developed QCL ellipsometer, depicted in Figure 1a, employs a spectrally broadband external cavity QCL (Hedgehog, DRS Daylight Solutions, San Diego, CA, USA) as an MIR light source. Operated in continuous wave mode, the laser emits highly monochromatic (spectral line width: ≤$3.3\times 10{⁻}^{3}$ cm^−1^) linearly polarized radiation in TEM_00_ spatial mode (beam waist: 2.5 mm, 1e2 intensity radius; beam divergence: < 4 mrad, full angle) with a maximum power of 110 mW according to the manufacturer. The external cavity tuning mechanism allows tuning the wavelength from 900 cm^−1^ to 1204 cm^−1^ (8.3 μm– 11.1 μm) at a speed of up to 1000 cm^−1^ per second, its wavelength repeatability (≤0.1 cm^−1^), accuracy (≤±0.5 cm^−1^) and precision (≤0.2 cm^−1^) guarantees excellent spectral resolution [37]. For alignment purposes, the QCL radiation passes two uncoated gold mirrors before it is transmitted through a set of two wire-grid polarizers on Si substrates (). The second polarizer ensures a well-defined polarization in the subsequent beam path; further, the set of both allows dimming the intensity in order to avoid destruction of the sample. The subsequent telescope configuration consists of two ZnSe lenses with diameters of 1 inch and ½ inch and focal lengths of 75 mm and 15 mm, respectively. At the cost of increasing beam divergence, it reduces the beam diameter by a theoretical factor of 5 before the polarization of the laser beam is modulated by means of a ZnSe photo-elastic modulator (PEM; PEM-90, Hinds Instruments, Hillsboro, OR, USA) operating at 37 kHz. While the optical axis of the PEM is rotated 45${}^{\circ }$ with respect to the incoming vertical polarization, the whole device is tilted about 30${}^{\circ }$ with respect to the beam path. Combined with the reduced beam diameter, this configuration enables us to spatially split the transmitted beam from multiple reflections occurring inside the PEM, block the latter by means of the following razor blade and therefore avoid modulated interference effects. The slightly divergent polarization-modulated beam is then projected onto the sample. The beam profile at the sample position was recorded for a wavelength of 1052 cm^−1^ by means of a microbolometer focal plane array (FPA; Boson, FLIR Systems, Wilsonville, OR, USA) and is depicted in Figure 1b. A Gaussian fit revealed a full width at half maximum of 1.4 mm and an almost perfect Gaussian profile. After transmission through the sample, the beam passes a third Si wire grid polarizer which acts as the polarization analyzer and is oriented parallel to the optical axis of the PEM. Finally, a ZnSe/ZnS achromatic doublet lens focuses the—now linearly polarized—radiation onto a thermoelectrically cooled mercury cadmium telluride (MCT) detector (PCI-4TE-12, VIGO System, Ożarów Mazowiecki, PL).

The analysis of the modulated detector signal is based on a reported procedure using lock-in amplification [12,38]. For this purpose, a signal extraction unit consisting of a digital multi-channel lock-in amplifier (eLockIn 204, Anfatec Instruments, Oelsnitz/Vogtland, Germany), high-pass and low-pass filters and a 12 bit high-speed oscilloscope (HDO6104A, Teledyne LeCroy, Chestnut Ridge, NY, USA) have been implemented for data acquisition. Following the cited approach, the acquired signals can be directly translated to the ellipsometric parameters Δ and Ψ, which describe the phase shift and amplitude ratio of two orthogonal polarization components introduced by the sample, respectively. Regardless of the type of ellipsometer applied, these two parameters can be exploited to access numerous material properties by means of data modeling and advanced evaluation methods. However, a QCL ellipsometer of this particular arrangement brings several important benefits. In addition to the high spectral brightness of a QCL, which results in an excellent SNR, the QCL emission enables collimated beams with diameters in the low millimeter range as well as essentially diffraction limited spot sizes using focusing optics—both unfeasible with conventional IR ellipsometers. Due to the fact that external cavity QCLs provide monochromatic but tunable emission, ellipsometers of this kind do not require any spectrometer or monochromator. While the combination of a PEM and the external cavity tuning mechanism allows sub-second time resolution, the latter in particular enables outstanding flexibility. Since the tuning range can be flexibly defined without any restrictions within the accessible wavelength range, observation of specific absorption bands or single-wavelength operation is possible—both reducing acquisition time much further below 1 s.

### 2.2. Polymer Thin Films and Multilayer Films

#### 2.2.1. Polyethylene/Ethylene Vinyl Alcohol Multilayer Film

Ethylene vinyl alcohol (EVOH) is a flexible oxygen barrier material with extensive use, particularly in food packaging. It is especially relevant for shelf-stable foods where oxygen deteriorates the quality of the products and shortens their shelf life [39,40]. In packaging materials, EVOH is often implemented as an intermediate layer in multilayer polymer films, for example, as oxygen barrier between polyethylene (PE) layers. Here, we demonstrate the ellipsometric investigation of a PE/EVOH/PE multilayer film with a total thickness of 116.5 μm and an EVOH oxygen barrier of 4.5 μm.

#### 2.2.2. Biaxially Oriented Polyethylene Terephthalate Thin Film

Polyethylene terephthalate (PET) is a thermoplastic polymer with numerous applications in packaging, fibers, injection molding and thin films. Biaxially oriented PET (BOPET) is manufactured by extruding a film of molten PET onto a chill roll, to quench it into the amorphous state and subsequently or simultaneously drawing it transverse to the rolling direction [41]. BOPET films are known for high tensile strengths, chemical and dimensional stability, transparency, gas and aroma barrier properties as well as electrical insulation [42,43]. In this contribution, we investigate the molecular structure of a 2.5 μm thin BOPET film (Mylar® Thin-Film, Chemplex Industries, Inc., Palm City, FL, USA) during a stretching process by means of QCL ellipsometry.

## 3. Results

QCL ellipsometry was employed to investigate the molecular structure and reorientation of a BOPET thin film during a stretching process. Additionally, a PE/EVOH/PE multilayer film film was examined under continuously varying angle of incidence. Since the data analysis, modelling and interpretation of ellipsometry spectra are entirely independent of the applied instrument—whether QCL based or conventional FTIR ellipsometer—we limit data analysis to a minimum and focus strictly on the significant benefits provided by QCL based instrumentation. A profound guideline on data modeling of the investigated structures—generally uniaxial anisotropic layers between isotropic ambient—can be found in [44].

### 3.1. Investigation of a PE/EVOH/PE Multilayer Film

Spectroscopic ellipsometry measures the phase shift Δ and amplitude ratio Ψ of two orthogonal polarization components, that are introduced by reflection at or transmission through a sample. While ellipsometric measurements in reflection configuration are most sensitive to surfaces, in transmission, the signal is dominated by features of the bulk material. Although the following discussion also applies to measurements in reflection, in this contribution, we limit ourselves to transmission ellipsometry. At oblique angle of incidence, a change in polarization indicates differing Fresnel transmission coefficients, which describe the transmission of light polarized parallel (p-polarized) and normal (s-polarized) to the plane of incidence. At normal incidence, however, there is no definition of a plane of incidence and the Fresnel transmission coefficients of isotropic media are degenerate. Thus, a measured change in polarization due to transmission at normal incidence directly accounts to anisotropic absorption, which in the case of polymer films can be interpreted as a predominant molecular orientation.

**Figure 2:**
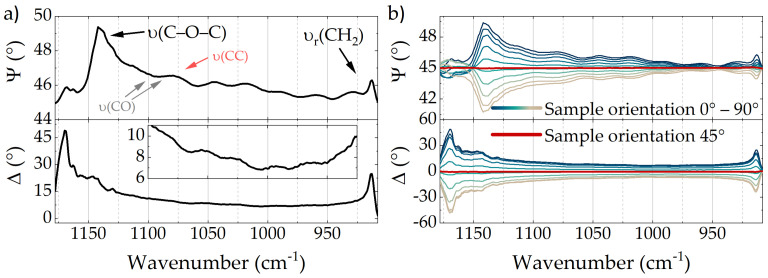
(**a**) Δ,Ψ-spectra of a 116.5 μm PE/EVOH/PE multilayer film recorded at normal incidence in transmission. The inset shows a magnification of the Δ-spectrum in the respective spectral range. Band assignment for PE (red) and EVOH (black/grey) according to literature [45,46]. (**b**) Spectra of the multilayer film recorded at normal incidence and different sample rotation around the beam path in 887 ms each.

Ellipsometry spectra of a 116.5 μm PE/EVOH/PE multilayer film recorded with the QCL ellipsometer in transmission at a normal incidence are depicted in Figure 2a. The acquired data featured a spectral resolution of 1 cm^−1^ before a 4 cm^−1^ moving average filter was applied to smooth the spectra. While the Ψ-spectrum shows several bands, which were assigned according to Liang and Krimm [45,46], the Δ-spectrum represents its Kramers–Kronig related counterpart. Except for the prominent ν(C-O-C) and $\nu_{r}\left(CH_{2}\right)$ bands the assigned bands can not be distinguished from the distinctive interference fringes observed—ν(CC) and ν(CO) bands are therefore only assigned for the sake of completeness. Due to the mentioned characteristics of ellipsometry at normal incidence, the presence of the ν(C-O-C) and $\nu_{r}\left(CH_{2}\right)$ bands reveals a predominant orientation of the associated molecular chains. Since, the Ψ-spectrum strictly shows positive bands for of all of the present bands we can conclude an orientation of the respective molecular chains along the *same* direction. To illustrate the dependence of the Δ,Ψ-signals with respect to the sample orientation, Figure 2b shows normal incidence measurements of the PE/EVOH/PE multilayer film in which the sample was rotated along the beam path. As depicted, maximum (positive bands) and minimum (negative bands) Δ,Ψ-spectra are observed at exactly the angles at which the anisotropy axes of the sample coincide with the xy-coordinate system of the instrument (0${}^{\circ }$ and 90${}^{\circ }$). At angles in between, however, only the projection onto the instruments xy-coordinates is measured. A rotation angle of 45${}^{\circ }$ leads to equal projections on the x- and y-axis and therefore to flat spectra without ellipsometric signals. An easy approach to determine the predominant molecular orientation is therefore to find the rotation angle of the flat spectra and rotate the sample additional $\pm 45{}^{\circ }$. The actual molecular orientation is then identified by means of the Δ,Ψ-amplitudes. While Ψ-signals > 45${}^{\circ }$ indicate a predominant alignment along the x-axis of our instrument, Ψ-signals < 45${}^{\circ }$ would indicate bands predominantly aligned along the orthogonal y-axis. Using QCL ellipsometry, this procedure can be monitored with a time resolution of 0.89 s for full spectra. If specific bands or even single wavelengths are examined, the time resolution can be considerably reduced to < 100 μs, which makes this technique perfectly fitting for in-situ applications.

**Figure 3:**
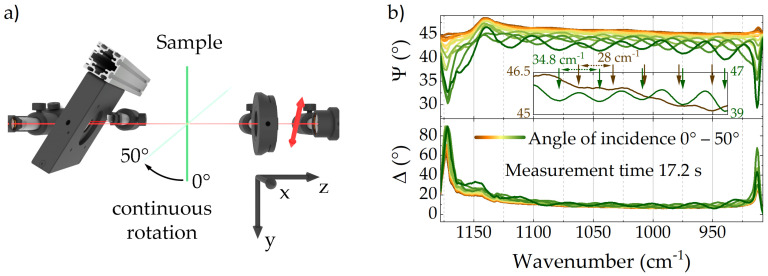
(**a**) Schematic of an IR-VASE measurement at continuously varying AOI. (**b**) Δ,Ψ-spectra of a 116.5 μm PE/EVOH/PE multilayer film recorded at continuously varying AOI. Frequencies of interference pattern for spectra at 0${}^{\circ }$ (brown) and 50${}^{\circ }$ AOI (green) are indicated in the inset.

Besides the assigned absorption bands, the Ψ-spectra shown in Figure 2 feature prominent interference fringes, which are especially noticeable in the spectral range from 930 cm^−1^ to 1080 cm^−1^. Kramers-Kronig related fringes can also be observed in the inset of Figure 2a, which illustrates the magnified Δ-spectrum in the respective spectral range. These fringes can be further exploited to determine film thickness and complex refractive index of the probed material by means of varying angle spectroscopic ellipsometry (VASE), in which Δ,Ψ-spectra are recorded for specific angles of incidence (AOI)—as indicated in Figure 3a. Since ellipsometry spectra relate to different Fresnel coefficients for p- and s-polarized light, which in turn depend on the AOI, wavelength and complex refractive index, modeling of the measured spectra using known parameters can provide information on unknown quantities. However, while in time-consuming conventional IR VASE measurements Δ,Ψ-spectra are typically recorded only for specific AOIs, the exceptional acquisition time of QCL ellipsometry allows real-time monitoring of sample properties during continuously varying geometry. To illustrate the accompanying opportunities, Figure 3b depicts a total of fourteen Δ,Ψ-spectra, which were recorded while the AOI was continuously increased from 0${}^{\circ }$ to 50${}^{\circ }$. The sample was initially aligned with its predominant molecular orientation parallel to the instruments coordinate system. As indicated, the phase and frequency of the interference pattern varies depending on the AOI. While the first Ψ-spectrum at 0${}^{\circ }$ AOI features a period of interference minima of 28 cm^−1^, for the last Ψ-spectrum at 50${}^{\circ }$ AOI this period was expanded to 34.8 cm^−1^ (Figure 3b inset). Assuming the observed interference is dominated by reflections at the interfaces with ambient air and neglecting refraction index changes due to the narrow EVOH layer, the period of interference minima $p_{min}$ of 28 cm^−1^ at oblique incidence is directly related to the overall film thickness *d* and the refractive index *n* of PE according to $d=\frac{1}{2n}\;\cdot \;\frac{1}{p_{min}}$. Given a known film thickness of 116.5 μm and solving with respect to *n* leads to an averaged refractive index of 1.53, which is in superb agreement with literature [47]. Taking spectra acquired at different AOI into account—by means of data modeling—can drastically increase information and provide access to additional parameters. However, since further data analysis is completely independent of the type of ellipsometer applied, we solely accentuate the unrivaled acquisition time of 17.2 s for the entire IR VASE measurement. This is particularly remarkable, since comparable measurements with a conventional FTIR ellipsometer would take multiple hours up to days [12].

### 3.2. Bopet Thin Film Stretching

**Figure 4:**
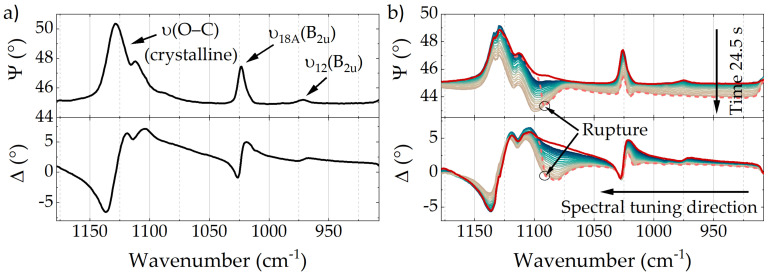
(**a**) Δ,Ψ-spectra of a 2.5 μm BOPET film recorded in a normal incidence transmission measurement. Band assignment according to literature [48]. (**b**) Ellipsometric monitoring of a BOPET film during stretching. The experiment took 24.5 s until the film tore during acquisition of the dashed spectra. While the blueish to brownish graphs were recorded during stretching, the red graph indicates the spectra after rupture.

Ellipsometry spectra of a 2.5 μm thin BOPET film recorded with the QCL ellipsometer in transmission at normal incidence are depicted in Figure 4a. The Ψ-spectrum features several bands, which were assigned according to Liang and Krimm [48]. Due to the mentioned characteristics of ellipsometry at normal incidence, the presence of the bands in the depicted spectra shows a predominant molecular orientation. Similar to the investigated multilayer film, all present bands are oriented along the same direction. A potential ν(O–C) vibration of amorphous PET at 1100 cm^−1^ observed by Liang and Krimm is hardly noticeable [48].

Figure 4b illustrates 23 ellipsometry spectra continuously acquired over the course of a stretching experiment of the BOPET film which lasted 24.5 s until the film tore. For this experiment the investigated film was fixed between two metal clamps with the predominant molecular orientation aligned perpendicular to the stretching direction. To gradually increase the tension applied to the film, the distance between the clamps was continuously increased by actuating a mechanical stage. While the blueish to brownish graphs were recorded during the ongoing stretching process, the dashed and red graphs were acquired during and after rupture, respectively. The former explicitly reveal the molecular reorientation within the film. Since the Ψ-signals decrease successively, a reorientation along the stretching direction (against the predominant direction) is indicated. Especially interesting is the increasingly strong presence of the mentioned ν(O–C) band of amorphous PET at 1100 cm^−1^, which appears to be predominantly oriented in the orthogonal direction (Ψ < 45${}^{\circ }$) already at moderate tensile strengths.

After increasing the applied force for 24.5 s, the BOPET film partially tore close to one of the clamps. On the one hand, the applied tension was relieved immediately after the rupture; on the other hand, since the film only partially tore, it was still fixed between the clamps and further recording of ellipsometric spectra was feasible. The spectra recorded when the film tore is depicted as dashed graph in Figure 4b. Since the emitted wavelength was tuned from low to high wavenumbers, the rupture is clearly noticeable in the recorded spectra. At wavenumbers > 1092 cm^−1^—which designate the moment of rupture—the dashed spectrum considerably differs from the one recorded previously. However, at these wavelengths the dashed spectrum coincides with the one recorded after the rupture, which is depicted in red. As indicated, at the moment of rupture—and relief of tension—the predominant molecular orientation snaps back to its initial state and the red spectra recorded after stretching coincide with the blue ones recorded at the beginning of the experiment. The only exception is the ν(O–C) band of amorphous PET at 1100 cm^−1^, which seems magnified after the stretching process. This experiment, especially the spectrum acquired during rupture of the film, excellently illustrates the ellipsometric investigation of fast dynamic processes in polymer thin films with unprecedented time resolution in the MIR. Since this technique is neither limited to stretching processes nor thin films, nor to transmission measurements, numerous applications in polymer processing are now conceivable.

## 4. Discussion

The benefits of QCL ellipsometry as a new method for advanced real-time in-situ monitoring applications in polymer processing have been demonstrated by studying different polymer thin films. First, a PE/EVOH/PE multilayer film was analyzed in transmission at a normal incidence to demonstrate the possibility to detect the predominant orientation of the molecular chains. Furthermore, the ability to acquire high-resolution broadband MIR ellipsometry spectra in less than 1 s was used to perform IR VASE measurements of the same film at a continuously varying incidence angle from 0${}^{\circ }$ to 50${}^{\circ }$. Those measurements took only 17.2 s. Since comparable measurements with conventional FTIR ellipsometers require acquisition times of multiple hours up to days; the reported experiment illustrates an unprecedented sample throughput for MIR ellipsometry. In addition, the dynamic molecular reorientation in a 2.5 μm thin BOPET film was investigated during a stretching process within 24.5 s. Besides insights into the molecular structure of the investigated thin film, this experiment revealed the potential of QCL ellipsometry for in-situ applications in the field of polymer processing.

Although a detailed modelling of the recorded spectra was not in the focus of this contribution—since it is completely independent of the applied type of ellipsometer—the large amount of sample parameters accessible with ellipsometry is evident from the data presented. The fact that the enormous versatility of spectroscopic ellipsometry is now accompanied by exceptional time resolution in the MIR opens up a whole new set of potential applications. For example, layer thicknesses, roughness and complex refractive index, especially in the MIR, and chemical information and molecular orientation are now accessible within less than 1 s. Targeting a specific question, the acquisition time can be further reduced by restricting the spectral range, for example to < 100 μs for single wavelength measurements. These unique features collectively qualify QCL ellipsometry as a suitable metrology for advanced in-situ monitoring especially in the field of polymer processing.
